# Crystal structures of *Leptospira interrogans *FAD-containing ferredoxin-NADP^+ ^reductase and its complex with NADP^+^

**DOI:** 10.1186/1472-6807-7-69

**Published:** 2007-10-24

**Authors:** Alessandro S Nascimento, Daniela L Catalano-Dupuy, Amanda Bernardes, Mario de Oliveira Neto, Maria Auxiliadora M Santos, Eduardo A Ceccarelli, Igor Polikarpov

**Affiliations:** 1 Instituto de Física de São Carlos, Universidade de São Paulo, Av. Trabalhador Saocarlense 400, São Carlos, SP, 13560-970, Brazil; 2 Facultad de Ciencias Bioquímicas y Farmacéuticas, Molecular Biology Division, Instituto de Biología Molecular y Celular de Rosario (IBR), CONICET, Universidad Nacional de Rosario, Suipacha 531, S2002LRK Rosario, Argentina

## Abstract

**Background:**

Ferredoxin-NADP(H) reductases (FNRs) are flavoenzymes that catalyze the electron transfer between NADP(H) and the proteins ferredoxin or flavodoxin. A number of structural features distinguish plant and bacterial FNRs, one of which is the mode of the cofactor FAD binding. *Leptospira interrogans *is a spirochaete parasitic bacterium capable of infecting humans and mammals in general. *Leptospira interrogans *FNR (LepFNR) displays low sequence identity with plant (34% with *Zea mays*) and bacterial (31% with *Escherichia coli*) FNRs. However, LepFNR contains all consensus sequences that define the plastidic class FNRs.

**Results:**

The crystal structures of the FAD-containing LepFNR and the complex of the enzyme with NADP^+^, were solved and compared to known FNRs. The comparison reveals significant structural similarities of the enzyme with the plastidic type FNRs and differences with the bacterial enzymes. Our small angle X-ray scattering experiments show that LepFNR is a monomeric enzyme. Moreover, our biochemical data demonstrate that the LepFNR has an enzymatic activity similar to those reported for the plastidic enzymes and that is significantly different from bacterial flavoenzymes, which display lower turnover rates.

**Conclusion:**

LepFNR is the first plastidic type FNR found in bacteria and, despite of its low sequence similarity with plastidic FNRs still displays high catalytic turnover rates. The typical structural and biochemical characteristics of plant FNRs unveiled for LepFNR support a notion of a putative lateral gene transfer which presumably offers *Leptospira interrogans *evolutionary advantages. The wealth of structural information about LepFNR provides a molecular basis for advanced drugs developments against leptospirosis.

## Background

Ferredoxin-NADP(H) reductases (FNRs) are flavoenzymes that catalyze electron transfer between NADP(H) and the iron-sulfur protein ferredoxin (Fd) or flavin mononucleotide-containing flavodoxin [1]. These enzymes are normally present as monomeric proteins in plastids, bacteria, mitochondria, and apicoplasts of intracellular parasites, where they catalyze the reaction described in equation (1), using noncovalently bound FAD as a prosthetic group.

2 Fd(Fe^+2^) + NADP^+ ^+ H^+^⇆2 Fd(Fe^+3^) + NADPH

Depending on the primary energy source of the organism the reaction catalyzed by FNR can be driven toward one of the directions indicated by the double arrow in equation (1). In heterotrophic bacteria and eukaryotes, the catalysis is driven toward ferredoxin reduction providing reducing power for a wide range of metabolic pathways [1,2].

Structural information is available for FNRs from plastids (maize leaf [3], spinach [4], pea [5]), cyanobacteria (*Anabaena *[6]) and bacteria (*Azobacter vinelandii *[7], *Escherichia coli *[8] and *Rhodobacter capsulatus *[9]). Topologically, there is a number of similarities within the two structural domains of FNRs of a different origin. The protein core, composed of a αβ-sandwich with a 5-strand β-sheet surrounded by 5 α-helices, is strongly conserved among FNRs. Structural differences however, appear in the size and conformation of the loops on the protein surface [10]. FAD binding site is located between the protein domains and has been described in detail by Bruns & Karplus [4].

The binding of the prosthetic group to FNRs from different sources is structurally different. The crystal structures of enzymes from plastids and cyanobacteria have FAD molecule in an extended conformation, while those from others prokaryotes are bended toward themselves and establish hydrogen bond interactions between the adenine and the isoalloxazine [1,11].

**Figure 1:**
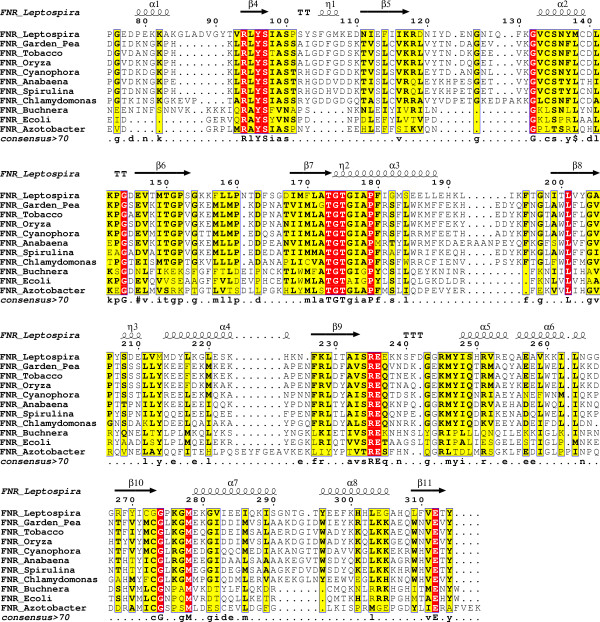
**FNRs sequence alignment**. Sequence alignment of plant and bacterial FNR. Identity is highlighted by color intensity. Alignment was performed in MUSCLE[36] and figure prepared in ESPript[37]. The arrows in the top denote sheet regions in the structure and the coils represent helices in LepFNR structure.

Sequence alignments of FNRs from different organisms revealed conservation profiles in close agreement with above discussed structural differences. A sequence alignment of plants, bacteria and algae FNRs reveals that the overall homology between enzyme primary structures is not so high, although amino acid sequence conservation between plant enzymes, for example, is significantly better (Fig. 1).

*Leptospira interrogans *is a spirochaete parasitic bacterium known to infect humans and mammals including cattle, dogs, pigs, horses and its natural carrier hosts, rodents. Quite surprisingly, according to phylogenetic analysis, *Leptospira *FNR (LepFNR) has been recently suggested to belong to the plastidic class FNRs [1]. The low identity in primary structure with the members of plastidic FNRs (Fig. 1), makes LepFNR an interesting target for structural and biochemical analysis aiming to better understand the structural grounds of the high catalytic efficiency of these oxidoreductases. In addition, the structure of LepFNR provides structural basis for the design of efficient and specific drugs dedicated to leptospirosis treatment.

In this work we submitted LepFNR to both structural and biochemical analysis. The crystal structures of LepFNR, presented here, reveal the molecular basis of recognition and binding of both prosthetic group FAD and substrate NADP^+ ^by the enzyme. Structural comparison of LepFNR with other FNRs, both from plant and bacterial origin, demonstrated more structural similarities of LepFNR with plant enzymes than with the bacterial FNRs. Small angle scattering experiments prove that LepFNR is a monomeric enzyme in solution. Finally, our biochemical data indicate that the LepFNR has a high enzymatic activity similar to those reported for the plastidic class enzymes and, considerably different from bacterial flavoenzymes which display lower turnover rates.

## Results and discussion

### Overall quality of the X-ray models

**Figure 2:**
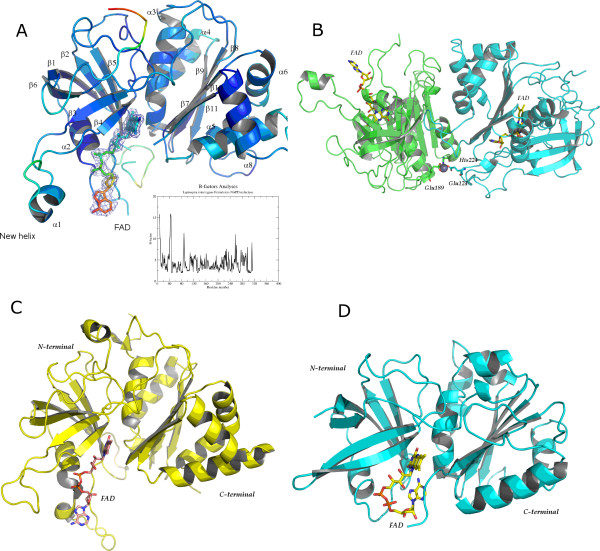
**FNRs Crystallographic models**. (a) Crystal structure of LepFNR colored by crystallographic B-factors indicates the region of flexible loops. 2F_obs_-F_calc _electron density map contoured at 1.0 sigma is shown for FAD molecule. In the insert, the plot of average B-factor per residue in LepFNR. (b) Detailed view of the metal binding site between two chains of LepFNR. The metal was modeled as a zinc atom. (c) Crystal structure of *Zea mays *FNR (PDB entry 1JB9[23]), showing FAD molecule in the extended form. (d) Crystal structure of *Azobacter vinelandii *(PDB entry 1A8P[7]), showing FAD molecule in bended form. The figures were prepared using PYMOL[38].

The quality of the LepFNR crystal structure, refined to 2.4 Å resolution, is fine as judged by stereochemical and refinement parameters (Table 1). Overall topology of the LepFNR molecule has the canonical FNR fold [10], which consists of two well defined domains, although several unique structural features were found in LepFNR 3D structure. The N-terminal domain forms a 6-stranded antiparallel β-barrel, and harbors the FAD binding site. The C-terminal domain folds as a αβ-sandwich with a 5-stranded β-sheet surrounded by six α-helices (Fig. 2). A unique feature present in LepFNR structure is the small helix formed in the loop 76–91 (highlighted in Fig. 2). This region has been previously identified as an important docking site of ferredoxin binding, mediated by its positively charged amino acid residues [11]. Although being exposed to solvent, this protein-protein recognition site has low mobility as judged from B-factors analyses (Fig. 2). Highly flexible regions of the protein are mostly confined to the surface loops (Fig. 2a).

**Table 1:** X-ray diffraction data and refinement statistics

	**LepFNR**	**LepFNR·NADP**^+^
Space group	P2_1_	P2_1_
Images collected (Δφ)	138 (1°)	196 (1°)
Wavelength (X-ray source)	1.42 Å (MX1-LNLS)	1.54 Å (Rotating anode)
Unit cell parameters	a = 65.11, b = 111.83, c = 89.91 (Å); β = 92.76°.	a = 67.16, b = 112.25, c = 92.39 (Å); β = 93.19°.
Resolution	53.92-2.43 (2.55-2.43) Å	35.65-2.70 (2.85-2.70) Å
Number of reflections	133681	106433
Number of unique reflections	47770	34663
Completeness	97.5% (97.5%)	92.2% (92.2%)
Redundancy	2.8 (2.6)	3.1 (2.5)
R_merge_	0.049 (0.144)	0.097 (0.434)
R_pim_	0.035 (0.110)	0.063 (0.293)
I/σ (I)	5.9 (2.3)	11.7 (2.3)
Total number of atoms	10229	10051
R_factor_	0.2113	0.261
R_free_	0.2792	0.297
RMS bond lengths	0.007 Å	0.015 Å
RMS bond angles	0.869°	1.829°

Values in brackets represent the values for each parameter in the highest resolution shell.

R_merge _= Σ_hkl _Σ_i _I_i_(hkl) - ⟨I(hkl)⟩/Σ_hkl _Σ_i _I_i_(hkl)

R_pim _= Σ_hkl _[1/(N-1)]^1/2 ^Σ_i_|I_i_(hkl) - ⟨I(hkl)⟩|/Σ_hkl _Σ_i _I_i_(hkl), where N is the redundancy measured.

Two out of the four molecules present in asymmetric unit of LepFNR crystal jointly coordinate a metal ion *via *two glutamates and one histidine residues, as shown in Figure 2b. This metal binding site is unique and has not been previously observed in any other FNR crystal structures. FNRs are known to function as monomeric proteins [11], however it has been recently suggested that two FNR isoforms from *Arabidopsis thaliana *are capable of dimer formation [12]. Thus, we decided to investigate the oligomeric state of LepFNR in solution by small angle X-ray scattering.

### LepFNR solution scattering

**Figure 3:**
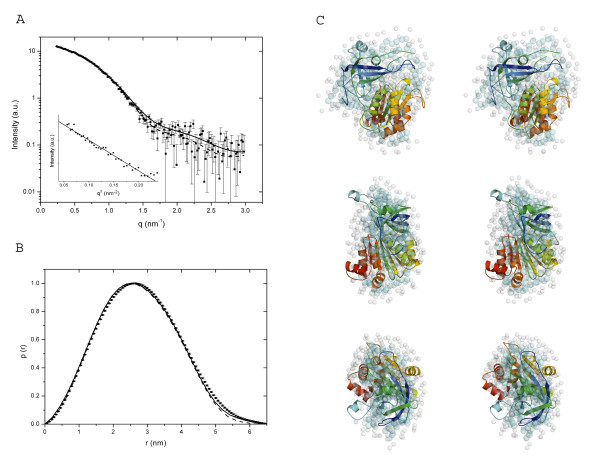
**LepFNR SAXS data**. (a) Experimental solution scattering curve of LepFNR and theoretical scattering intensities. Desmeared experimental curve is shown as dots with error bars; theoretical scattering intensity from the DAM is given in continuous line; scattering intensity from the LepFNR monomer is shown as a broken line. An inset displays the correspondent Guinier plot. (b) Comparison of distance distribution functions for LepFNR. The p(r) curves obtained were calculated using the GNOM program, the experimentally derived distribution for LepFNR is shown as dots with error bars. Distributions were calculates for the DAM (continuous line) and a single monomer of LepFNR crystal structure (broken line). (c) Stereoviews showing the superposition of the high-resolution crystallographic monomer of LepFNR with the envelope obtained from the data SAXS (spheres). Middle and bottom images are rotated by 90° around the y and x axes, respectively, compared to the top image.

X-ray scattering curves at the protein concentration of 3 mg/ml and 10 mg/ml have essentially the same profile; therefore we used scattering data measured at 10 mg/ml to benefit from their better statistics and resolution. Comparison of SAXS data with crystallographic structures of LepFNR monomers and dimers using CRYSOL reveals that LepFNR forms monomers in solution (Fig. 3a and Table 2). Analysis of the distance-distribution *p*(*r*) function (Fig. 3b) led us to conclude that the protein has a maximum dimension Dmax of 6.50 ± 0.50 nm, compatible with a FNR monomer. The value of 2.13 ± 0.50 nm for Rg calculated from the distance distribution function is in a good agreement with the estimate derived from the Guinier analysis of 2.16 nm. Furthermore, a structural superposition of the *ab initio *low-resolution envelope of the protein with the high-resolution X-ray structure of the LepFNR monomer (Fig. 3c) leaves no doubt that the protein is indeed a monomer in solution.

**Table 2:** Structural parameters obtained from SAXS data

Parameters/Sample	LepFNR		
	
	Exp. *	Mod. †	DAM ‡
D_max _(nm)	6.50 ± 0.50	6.14	6.00
R_g _(nm)	2.13 ± 0.50	1.93	1.97
Free parameters	5.00^€^	-	263.00^ζ^
Discrepancy χ	-	1.18^χ^	1.10^χ^
Volume (nm^3^)	-	47.30	50.70
Resolution (nm)	2.11^¶^	-	2.11^¶^
MW (kDa)	34.24^η^	-	-

* Exp., calculated from the experimental data at 10 mg/ml.

† Mod, parameters obtained from the LepFNR monomer crystallographic structure

‡ DAM, parameters of the averaged dummy atom model over 10 models.

€ Shannon channels number N_s _= [D_max_(q_max_-q_min_)]/π

π Model residues number

χ Parameter of comparison with experimental data

¶Resolution = 2*π/q_máx_

η MW obtained by comparison with standard protein BSA

Our results on LepFNR monomeric form obtained from solution X-ray scattering diverge from the results of the recent crystallographic studies of *Plasmodium falciparum *FNR [13]. In the latter protein, two oxidized cysteine residues form a covalent bond joining two molecules in a dimer. The cysteine residues involved in the bridge is highly conserved over the plasmodial FNRs, but not in plant and homologous FNRs [13]. LepFNR, on the other hand, exhibits higher sequential identity with plant FNRs and does not contain cysteine residues in the position required to form the intermolecular disulfide bridge. This analysis strongly suggests that metal mediated LepFNR dimerization, observed in the crystal structure, is induced by crystallization effects and do not represent a physiologically relevant oligomeric state of the protein.

### FAD binding site

Crystal structure analysis reveals that unlike in usual prokaryote FNRs, LepFNR accommodates the FAD molecule in an extended conformation between the two enzyme domains (Figs. 2a, 2c and 2d). Adenosine portion of FAD is less restrained and more disordered as could be concluded from LepFNR crystallographic B-factors analysis. An increased disorder of adenosine portion of FAD as compared to the whole molecule is common for plastidic class FNRs [4].

**Figure 4:**
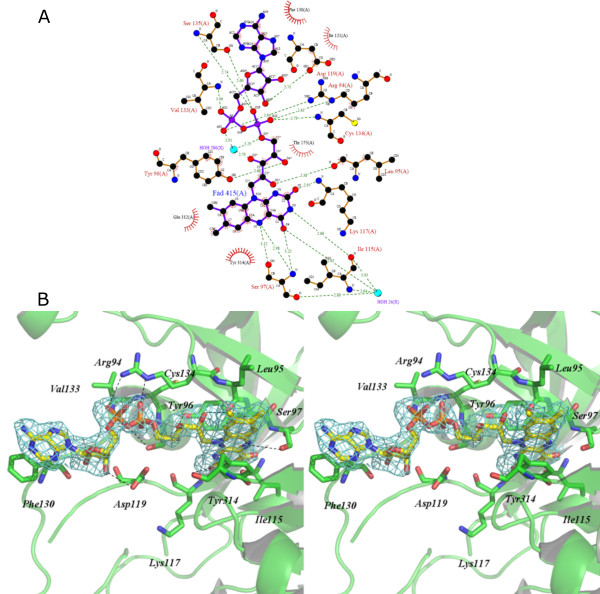
**FAD binding to FNR**. (a) Schematic representation of FAD binding to FNR, prepared using LIGPLOT[39]. (b) Stereo view of FAD binding site. Key residues in FAD binding are shown with sticks.

The flavin group of FAD is tightly bound to FNR binding cleft through strong hydrogen bonds (Figs. 4a and 4b). The main chain atoms of Ser97, Ile115, Lys117 and Leu95 interact directly with flavin moiety creating the core of the binding site (Fig. 4b). Ser97 and Tyr96 also interact to flavin group *via *their side chains, adding to a stereochemical environment able to bind and tightly fix the FAD molecule. It is interesting to note that although Ile115 plays an important role in flavin recognition and interacts with the flavin group through a strong hydrogen bond (2.66 Å in length), this residue is not conserved in FNRs (Fig. 1). Similarly, Lys117 is only partially conserved in FNR family and is present in approximately half of the FNRs shown in the alignment (Fig. 1). Crystal structure analysis reveals, however, that both residues participate in FAD recognition and binding through their main chain groups, and, therefore, evolutionary restraints on the specific amino acid type have been lost at these positions.

The adenosine group also participates in a rich hydrogen bond network through its pyrophosphate portion. Cys134, Arg94, Ser135 and Val133 are at hydrogen bonding distances to pyrophosphate group strongly anchoring it to LepFNR cleft. This results in a low mobility of adenosine group in LepFNR structure. Adenosine ribose is also involved in strong hydrogen bond interaction with Asp119 as judged from a short 2.71 Å length of this bond. This is a particular feature of LepFNR since this residue is not conserved over FNR family. At variance, this aspartate is replaced by a hydrophobic leucine residue in plastidic FNRs and by an alanine in some bacterial FNRs, so that this additional hydrogen bond in adenosine binding pocket appears to be new in FNR structures. The phenol ring of the carboxyl terminal tyrosine (Tyr314) and the flavin group are practically coplanar maximizing π-orbital overlapping, as have been observed in all plastidic class FNRs [11], resulting in a large portion of the isoalloxazine moiety shielded from the bulk solution. This structural feature, together with the extended FAD conformation, has been implicated in the high catalytic efficiencies displayed by the plastidic FNRs [1,11].

Structural comparison between plant and bacterial FNRs reveals an important and distinguished feature among them. Plastidic FNRs have a common motif SLCV(^K^/_R_)(^R^/_Q_)(^L^/_A_). In LepFNR this region is less conserved (113-EFIIKRDN-120) but still contains both basic amino acids (Lys and Arg) close to the FAD binding site. In all plastidic FNRs previously known, FAD is bound in an extended conformation and its adenine interacts with a tyrosine residue belonging to a typical cluster always present [1]. Surprisingly, in LepFNR FAD acquired a different conformation in which its adenine moiety interacts with Phe130 (Fig 4b). The latter amino acid is not conserved in other plastidic FNRs and resides on the protruding sheet-loop-sheet interacting with the 2'-P-AMP region of FAD. This structural motif is missing in all bacterial class FNRs. Moreover, in the bacterial enzymes the basic residues are replaced by the hydrophobic isoleucine and leucine residues that accommodate the bended FAD in the cleft between the N and C-terminal domains. Taken together this evidence provides an explanation why FAD molecule binds to LepFNR in an extended conformation, typical for plant FNRs, and not in a bended form observed in bacterial FNRs, further highlighting structural and sequence similarities between *Leptospira *FNR and its plant homologs.

### LepFNR·NADP^+ ^complex crystal structure

**Figure 5:**
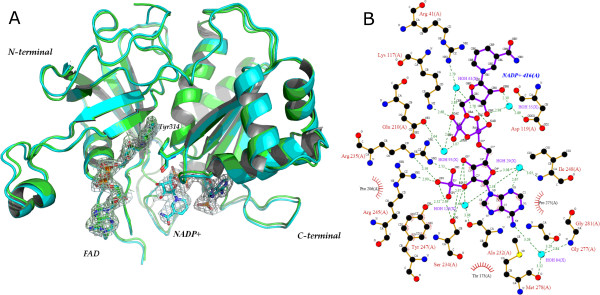
**Comparison of LepFNR crystallographic models bound to FAD and NADP^+^**. (a) Superposition of LepFNR (green) and LepFNR·NADP^+ ^(blue) crystal structures. Electronic densities are contoured at 1.0 sigma for FAD andNADP^+^. (b) Schematic representation of NADP^+ ^binding to LepFNR.

LepFNR·NADP^+ ^crystal structure, refined to 2.70 Å resolution, revealed moderate structural changes when compared to LepFNR crystal structure (Fig. 5a). Statistics of the model is given in Table 1. The high quality of electron density maps, allowed the unambiguously positioning of the NADP^+ ^molecule noncovalently bound to the C-terminal domain of LepFNR.

Small, but distinct, structural rearrangements could be identified upon NADP^+ ^binding. Tyr247 changes its side-chain conformation and bends toward the NADP^+ ^in LepFNR·NADP^+ ^crystal structure, thus facilitating interactions between adenine phosphate and its side chain hydroxyl group. This feature was also observed in the crystal structure of *Anabena *FNR [14]. NADP^+ ^binding also causes changes in Arg245 conformation, which also is part of the NADP^+ ^binding site (Fig. 5b). NADP^+ ^molecule is attached to LepFNR C-terminal domain by a network of hydrogen bonds (see Fig. 4b). Similarly to FAD binding, the adenine portion of NADP^+ ^is tightly bound to LepFNR. In opposite, the nicotinamide group has an increased mobility, as indicated by crystallographic B-factors. At atomic level, adenine moiety makes water-mediated hydrogen bonds with Gly277, Gly281, Met278 and Ile248 main chain atoms. The ribose group also interacts with Ser234 and forms water-mediated hydrogen bonds with Ala232. The phosphate group is anchored by the hydrogen bonds with Arg245 and Arg235. All residues mentioned above have their counterparts in the already identified crystal structures of pea and *Anabaena *FNRs complexed with NADP^+ ^[5,14]. At variance, in LepFNR the nicotinamide portion of NADP^+ ^makes water mediated hydrogen bonds with Asp119, Arg41 and Glu210. Lysine 117 also interacts to NADP^+ ^by a hydrogen bond with oxygen of phosphate group, as indicated in Figure 4b.

The overall structural adjustments induced by NADP^+ ^binding in LepFNR structure are modest, and RMSD deviations between NADP^+ ^bound and NADP^+^-free states are of 0.453 Å as calculated for Cα positions only. Structural superposition of the 3D models using their N-terminal domains shows a maximum displacement of 1.0 Å between respective Cα positions in these two models, which is confined to the C-terminal helices region. The superposition of the structures is given in Figure 5.

### Enzymatic activity

As shown in Table 3, LepFNR has a high diaphorase activity, reaction commonly used to characterize FNRs [11]. Similarly, LepFNR exhibited *K*_m _for NADP^+ ^which is identical to the one obtained for the pea (*P. sativum*) enzyme (Table 3). The *k*_*cat*_/*K*_m _for LepFNR is *circa *75% with respect to that of the latter FNR. These steady state kinetic studies reveal that the LepFNR enzyme has high affinity for NADPH. The affinity for NADP^+ ^was determined by difference absorption spectroscopy after successive addition of the nucleotide. The LepFNR enzyme has a *K*_d _for NADP^+ ^of 43 μM, which is in the same range of the affinities observed for the *P. sativum *enzyme and other reductases (Table 3 and ref [11]). These data are in agreement with the suggested affinities for NADPH deduced from steady state kinetic studies.

**Table 3:** Kinetic and binding parameters for LepFNR NADPH-ferricyanide diaphorase activity

FNR	*K*_d _(μM, NADP^+^)	*K*_m _(μM, NADPH)	*k*_cat _(s^-1^)	*k*_cat_/*K*_m _(μM^-1^·s^-1^)
*L. interrogans*	43 ± 3	19.5 ± 1.7	258 ± 13	13.2
*P. sativum*	32 ± 2	19.0 ± 1.6	324 ± 16	17.1

The different parameters were obtained as described in Experimental Procedures. Each parameter value represents the average of 3 independent experiments.

## Conclusion

Crystal structures of LepFNR reveal a typical plastidic type reductase, analogous in its folding architecture to the enzymes found in plant and cyanobacteria, in spite of displaying an important divergence at primary structure level. The typical FAD and NADP^+ ^binding domains are well conserved among them and the amino acids involved in prosthetic group and NADP^+ ^binding have a high degree of structural conservation. Our analysis reveal several distinct key features of this structure: 1) The FAD molecule is bound in an extended conformation and the adenosine moiety is interacting with a different aromatic residue than in all other plastidic reductases; 2) The poorly conserved amino acid fragment 113-EFIIKRDN-120 still contains both basic amino acids that participate in the FAD binding; 3) The carboxyl terminal amino acid is a tyrosine, that is planar to the isoalloxazine at 3.6 Å distance. All these features of the LepFNR are found in enzymatically highly efficient reductases and are in a good agreement with the biochemical data we have obtained for this enzyme.

Structural comparison between present LepFNR crystal structures and the X-ray structure of the previously determined *Anabaena *FNR complexed with NADP^+ ^[6,14] reveals a number of important similarities in the model of substrate binding between these two enzymes. The adenosine part of NADP^+ ^and the 2'phosphate group of the substrate are in equivalent positions in these two structures. In both models the nicotinamide reactive part of substrate is not positioned in a productive form and was found too far from the isoalloxazine to allow electron transfer [this work and ref 14]. The position observed for the *Anabaena *FNR·NADP^+ ^complex is somewhat different than the observed in LepFNR·NADP^+ ^crystal structure. A sequence of three steps for the coenzyme recognition and binding mechanism has been proposed to explain the different conformation obtained in the *Anabaena *FNR·NADP^+ ^complex [15]. Here, the position adopted by the NADP^+ ^nicotinamide bound to the LepFNR enzyme may represent another intermediate before the productive binding of the substrate.

An interesting outcome of our analysis is that the binding of the substrate to holoenzyme produces not only a rearrangement of some of the residues involved in its binding but also a moderate but indubitable conformational change in the carboxyl terminal domain. This structural rearrangement can help to explain the strong negative cooperativity that has been observed for the ferredoxin and NADP^+ ^binding [16].

Atomic coordinates and structure factors were deposited to the Protein Data Bank and received accession codes 2RC5 and 2RC6.

## Methods

### Protein expression and crystallization

LepFNR was expressed and purified as described [17]. Briefly, the vector pET32JO-LepFNR containing the DNA sequence for the protein fused with the thioredoxin DNA was expressed in *E. coli *grown at 37°C in LB medium, which was supplemented with ampicillin and chloranphenicol. Expression was induced with 1 mM IPTG for 3 hours at 30°C. LepFNR was purified by Ni-NTA affinity chromatography and dialyzed against 50 mM Tris-HCl buffer, pH 8.0 at the presence of 150 mM NaCl. The fusion protein was digested with thrombin and the thioredoxin removed by another Ni-NTA affinity chromatography procedure. Protein was concentrated by centrifugation in a Centripep-10 (Amicon) to a final concentration of 29 mg/ml.

After an initial crystallization screening, the crystals were grown in a condition with 30% PEG 3350, 0.3 M ammonium fluoride, pH 6.5 [17]. Better crystals were obtained in the optimized condition containing 27% PEG 3350, 50 mM Tris-HCl buffer pH 7.0 at 291 K. Crystals of LepFNR bound to NADP^+ ^were obtained by co-crystallization in conditions similar to those described above. NADP^+ ^was added prior to crystallization trials.

### Data collection and processing

A complete diffraction dataset from a single LepFNR crystal was recorded at MX1 beamline of Brazilian Synchrotron Light Laboratory (LNLS) [18,19]. The synchrotron radiation wavelength of 1.42 Å was chosen to optimize both the diffraction efficiency of the protein crystal and the synchrotron-radiation flux at this medium energy synchrotron [20,21]. A single crystal was transferred to 5 μl of the mother liquor supplemented with 1 μl ethylene glycol. The crystal was flash-cooled in a nitrogen stream at 100 K prior to data collection. Images were processed and integrated using MOSFLM [22]. Scaling was carried out with SCALA, from CCP4 suite [23].

The LepFNR·NADP^+ ^complex diffraction data was collected from a crystal flash-frozen following the same procedure. The dataset was collected at Rigaku Ultra X18 rotating anode, operating at Cu Kα wavelength radiation, which was equipped with Osmic Confocal mirror optics and a MAR345 image plate detector. Images were indexed, integrated and scaled using MOSFLM and SCALA (CCP4 suite). The statistics of data reduction for both crystals are given in Table 1.

### Structure solution and refinement

Attempts to solve LepFNR by molecular replacement were carried out using both *Anabaena *FNR (PDB entry 1OGJ[15]) and maize root FNR (PDB entry 1JB9[24]). Maize root FNR resulted in a better initial model and was used to further refinement. The structure was improved prior to molecular replacement using CHAINSAW (CCP4 suite) and employed as a search model in molecular replacement procedure using PHASER [25]. The molecular replacement was able to find four independent protein molecule positions in the asymmetric unit. An initial refinement cycle was performed with CNS [26] using simulated annealing procedure. Iterative cycles of model building and refinement were carried out using COOT [27], REFMAC [23] (CCP4) and PHENIX [28]. To decrease a number of free parameters, tight non-crystallographic symmetry between the four molecules in asymmetric unit was applied in all refinement steps. The crystal structure of LepFNR·NADP^+ ^was solved by molecular replacement using LepFNR refined structure. Statistics of the refined models are presented in Table 1.

### Small-angle X-ray scattering measurements and data analysis

SAXS is a fundamental tool in the study of biological macromolecules in solution, which permits to study the low-resolution structure of proteins in near physiological environments [29,30]. SAXS data for LepFNR molecular shape reconstruction were collected at the small-angle scattering beamline of the LNLS at the protein concentrations of 3 and 10 mg/ml. The wavelength of the incoming monochromatic X-ray beam was λ = 0.148 nm. A 1D X-ray position sensitive detector (PSD) was utilized to record the scattered intensity as a function of the modulus of the scattering vector *q *(*q *= 4·π·sin(θ/λ), where θ is half the scattering angle). The parasitic scattering from air and beamline windows was subtracted from the total measured intensities. The sample-to-detector distance (1155.1 mm) was adjusted in order to record the scattering intensity for *q *values ranging from 0.1 <*q *< 3 nm^-1^. Radii of gyration *R*g was evaluated by two methods, Guinier (ln I(q) x q^2^)^28 ^and by the indirect Fourier transform program GNOM [31]. The distance distribution function *p*(*r*) was also computed by this program. The molecular mass of the LepFNR in solution was estimated by comparison of the extrapolated forward scattering I(0) with that of a reference solution of bovine serum albumin with a known molecular mass of 66 kDa. Dummy Atom Models (DAMs) were generated *ab initio *using the program GASBOR [32]. The LepFNR crystallographic structure was superimposed onto the DAMs using SUPCOMB [33]. CRYSOL [34] was used for comparison of high-resolution models and the data derived from experiment.

### Enzymatic activity measurements

FNR-dependent diaphorase activity was determined by a published method [35]. The reaction mixture (1 ml) contained 50 mM Tris-HCl, pH 8, 3 mM glucose-6-phosphate, 0.3 mM NADP^+^, 1 unit of glucose-6-phosphate dehydrogenase, and 1 mM potassium ferricyanide. After the addition of ~20 nM LepFNR, the reactions were monitored spectrophotometrically by following potassium ferricyanide reduction at 420 nm (ε420 = 1 mM^-1^·cm^-1^). The experiments have been conducted at 30°C.

### Determination of the dissociation constant of the LepFNR·NADP^+ ^complex

Difference absorption spectroscopy was used to evaluate the dissociation constant of the LepFNR·NADP^+ ^complex. The experiment was performed essentially as previously described [2] with a solution containing 35 μM LepFNR in 50 mM Tris-HCl, pH 8, which was titrated at 25°C with the substrate.

## List of abbreviations

FNR, ferredoxin-NADP^+ ^reductase; NADP^+^, nicotinamide adenine dinucleotide phosphate; IPTG, isopropyl β-D-1-thiogalactopyranoside; SAXS, small angle X-ray scattering; FAD, flavin adenine dinucleotide.

## Competing interests

The author(s) declares that there are no competing interests. 

## Authors' contributions

EAC, DLC, ASN and IP conceived the study. ASN, DLC, MAMS, MON, AB prepared the samples and collected the data. ASN and DLC interpreted the data and wrote the paper. All authors read, modified and approved the final manuscript.
